# 38.2 NEURONAL AUTOANTIBODIES IN PSYCHOSIS: ENOUGH ABOUT PREVALENCE, WHAT’S THE RELEVANCE?

**DOI:** 10.1093/schbul/sby014.155

**Published:** 2018-04-01

**Authors:** Thomas Pollak

**Affiliations:** Institute of Psychiatry, Psychology & Neuroscience, King’s College London

## Abstract

**Background:**

One source of controversy in the emerging field of autoimmune psychiatry concerns varying prevalence estimates of neuronal surface autoantibodies (NSAbs) in psychiatric disorders, particularly psychotic disorders. Differences in assay methodology and patient selection may contribute to varying case-control estimates.

I will argue that the field needs to move beyond small n prevalence studies, to address the question of the relevance of NSAbs in psychotic disorders, and namely the following questions:

1) Does the presence of NSAbs offer any aetiopathological insights into psychosis i.e. by associating with other disease-relevant biomarkers?

2) Do NSAbs shape the clinical phenotype of psychotic disorders?

3) Do NSAbs have a predictive role in psychotic disorders, in terms of treatment response or course of illness?

**Methods:**

To address this issue, we have undertaken measurement of NSAbs using multiple immunoassays in a cohort of individuals at ultra-high risk for psychosis, and another first episode psychosis cohort. Associations between NSAb seropositivity and phenotype, outcome and biomarkers including structural MRI were explored.

**Results:**

NSAbs were detected at rates of between 1% and 9% of cases in both cohorts, depending on assay used. Live CBAs detected significantly more NMDAR and GABA-AR IgG antibodies than did fixed CBAs. Rates in cases were not significantly different from controls, regardless of assay.

Nevertheless in UHR subjects NSAbs, and NMDAR Abs in particular, showed clear aetiopathological and phenotypic relevance, associating with cognitive function (poorer verbal memory and IQ), more severe psychopathology and increased volumes of key limbic areas. Significant interactions with a marker of blood-brain barrier integrity offered further aetiopathological insights. NSAbs detected by both fixed and live CBAs demonstrated phenotypic associations and interactions with BBB status, suggesting both assays can detect phenotypically relevant antibodies in the UHR context. In FEP subjects, no such associations were noted. GABA-A receptor antibodies, which have been proposed as NSAbs with emerging disease-relevance, showed no phenotypic associations.

Data on the predictive utility of NSAbs in UHR and FEP subjects will be presented.

**Discussion:**

With appropriately fine-grained phenotyping and careful consideration of moderating biological factors and assay variation, clear disease-relevance of NSAbs could be established in UHR subjects but not in FEP subjects. In particular, NMDAR antibodies may have important biomarker potential in the at-risk mental state.

The failure to establish clear disease-relevance in previous psychiatric cohorts may reflect a genuinely irrelevant antibody but could also be due any of the following:

1) Inadequately fine-grained phenotyping of subjects

2) Ignoring the important moderating role of BBB permeability

3) Choosing subjects at ‘too late’ a stage of illness

4) Inadequately sensitive antibody detection assays

